# A Cardiovascular Disease Prediction Model Based on Routine Physical Examination Indicators Using Machine Learning Methods: A Cohort Study

**DOI:** 10.3389/fcvm.2022.854287

**Published:** 2022-06-17

**Authors:** Xin Qian, Yu Li, Xianghui Zhang, Heng Guo, Jia He, Xinping Wang, Yizhong Yan, Jiaolong Ma, Rulin Ma, Shuxia Guo

**Affiliations:** ^1^Department of Public Health, Shihezi University School of Medicine, Shihezi, China; ^2^Department of NHC Key Laboratory of Prevention and Treatment of Central Asia High Incidence Diseases, The First Affiliated Hospital of Shihezi University Medical College, Shihezi, China

**Keywords:** cardiovascular disease, machine learning, predictive models, routine physical examination indicators, cohort study

## Abstract

**Background:**

Cardiovascular diseases (CVD) are currently the leading cause of premature death worldwide. Model-based early detection of high-risk populations for CVD is the key to CVD prevention. Thus, this research aimed to use machine learning (ML) algorithms to establish a CVD prediction model based on routine physical examination indicators suitable for the Xinjiang rural population.

**Method:**

The research cohort data collection was divided into two stages. The first stage involved a baseline survey from 2010 to 2012, with follow-up ending in December 2017. The second-phase baseline survey was conducted from September to December 2016, and follow-up ended in August 2021. A total of 12,692 participants (10,407 Uyghur and 2,285 Kazak) were included in the study. Screening predictors and establishing variable subsets were based on least absolute shrinkage and selection operator (Lasso) regression, logistic regression forward partial likelihood estimation (FLR), random forest (RF) feature importance, and RF variable importance. The selected subset of variables was compared with L1 regularized logistic regression (L1-LR), RF, support vector machine (SVM), and AdaBoost algorithm to establish a CVD prediction model suitable for this population. The incidence of CVD in this population was then analyzed.

**Result:**

After 4.94 years of follow-up, a total of 1,176 people were diagnosed with CVD (cumulative incidence: 9.27%). In the comparison of discrimination and calibration, the prediction performance of the subset of variables selected based on FLR was better than that of other models. Combining the results of discrimination, calibration, and clinical validity, the prediction model based on L1-LR had the best prediction performance. Age, systolic blood pressure, low-density lipoprotein-L/high-density lipoproteins-C, triglyceride blood glucose index, body mass index, and body adiposity index were all important predictors of the onset of CVD in the Xinjiang rural population.

**Conclusion:**

In the Xinjiang rural population, the prediction model based on L1-LR had the best prediction performance.

## Introduction

Cardiovascular disease (CVD), a chronic and complex disease caused by heart and vascular diseases, is currently the main cause of premature death and chronic disability globally (1, 2). Its treatment usually involves medical and surgical methods. Nevertheless, these treatments cannot cure CVD. Moreover, these treatments have a great impact on the quality of life of individuals with CVD. Therefore, the current management of CVD mainly focuses on preventive measures. Recent studies suggest that ~80% of premature CVD mortality could be prevented through early intervention (3). In addition, CVD has a slow onset and long incubation period; thus, it is generally at a more serious stage at the time of diagnosis. Therefore, early identification of high-risk groups for CVD is particularly important for its prevention and control (4).

In recent years, an increasing number of CVD prevention and control guidelines recommended the use of CVD risk prediction models to identify high-risk groups who could receive early intervention to reduce CVD risk (5). Most current risk prediction models for CVD were established using traditional statistical methods (6–10). A model is established if it meets the requirements of independence and linearity. Therefore, it cannot reflect the complex relationship between variables, which affects the accuracy of the prediction model and the applicability of external verification (11, 12). The machine learning (ML) algorithm is a traditional statistical method that can effectively solve the problems of non-linearity, variable redundancy, and interaction between variables. Moreover, it can be used to explore the potential risk factors for CVD to improve its predictive performance; hence, it is widely used in the field of CVD prevention and control (13). Despite its advantages, there are still controversies regarding its ability to predict CVD. Related studies reported that the predictive performance of ML algorithms was better than those of traditional statistical methods (14). Contrastingly, studies showed that the predictive performance of logistic regression (LR) was not weaker than that of machine learning algorithms (15, 16).

Xinjiang is located in northwest China and is home to multiple ethnic groups. Uyghur and Kazakh are the main ethnic groups in Xinjiang. Studies found that these populations have high prevalence of CVD risk factors, such as metabolic syndrome, hypertension, and obesity, thereby corresponding with high incidence of CVD (17–20). Most prediction models for CVD are based on European and American populations (6, 9, 21). Although in recent years, Chinese researchers have established predictive models based on Cox regression and ML algorithms, most are based on a feature screening method for predictive modeling (22, 23). Moreover, there are few reports on ethnic minority groups in Xinjiang, and previous studies showed that the Framingham risk score (FRS) and Pooled Cohort Equations (PCEs) were not suitable for identifying groups that had a high risk of CVD among the Uyghur and Kazak populations (24).

Thus, this study aimed to use machine learning algorithms to establish a CVD prediction model that was suitable for the Xinjiang Uyghur and Kazak populations based on routine physical examination indicators. This study also aimed to identify the main factors that affect the occurrence of CVD, to identify groups that had a high risk of CVD in early-stage disease, to provide a theoretical basis for the effective prevention of CVD, and to have important, practical significance for the comprehensive prevention and control of CVD in the Uyghur and Kazak populations.

## Methods

### Study Population

Baseline data collection was divided into two phases. In the first stage, a baseline survey was conducted from 2010 to 2012. Through stratified cluster random sampling, the Uyghur population in Jiangbazi Township, Jiashi County, Kashi Prefecture, and southern Xinjiang, and the Kazakhs in Nalati Township, Xinyuan County, Ili Prefecture, and northern Xinjiang were selected. In the second stage, a baseline survey was conducted from September to December 2016, and the Uyghur population of the 51st Regiment of the Third Division of the Xinjiang Corps was selected as the research cohort through stratified cluster random sampling. A total of 19,549 people who were aged ≥18 years and lived in the local area for >6 months were included in the study. The exclusion criteria included CVD at baseline, those lost to follow-up, and those with incomplete blood information. Follow-up continued until December 2017 for the first stage (median: 6.07 years) and until August 2021 for the second stage (median: 4.94 years). According to the inclusion and exclusion criteria, 5,335 and 7,357 people were included in the first and second stages, respectively, for a total of 12,692 individuals (Supplementary Figures 1.1, 1.2). Then do data analysis (Supplementary Figure 1.3). All participants provided written informed consent. This study was approved by the Ethics Committee of the First Affiliated Hospital of Shihezi University School of Medicine (NO. SHZ2010LL01).

### Data Collection

Data were collected *via* questionnaire, physical examination, and laboratory examination. Questionnaires were completed face-to-face. Anthropometric measurements such as height, weight, waist circumference (WC), hip circumference (HC), and blood pressure were obtained by trained professionals. Blood pressure was measured three times for each participant using a mercury sphygmomanometer after 5-min seated rest, and the average value was calculated. Hypertension was defined as systolic blood pressure (SBP) of ≥140 mmHg or diastolic blood pressure (DBP) of ≥90 mmHg. Prehypertension was defined as 140 > SBP ≥ 120 mmHg or 90 > DBP ≥ 80 mmHg (25). Synthetic indices were calculated based on anthropometric measurements: BMI [weight (kg)/height^2^ (m)]; BAI (HC/height^1.5^-18); pulse pressure (SBP–DBP); and waist-to-hip ratio [WHR; WC (cm)/HC (cm)]. A family history of diabetes was defined as a history of diabetes in at least one parent or sibling; the same criteria were used for a family history of stroke and coronary heart disease (CHD). Current smokers were defined as participants who had been smoking for >6 months (26). Drinking was defined as consuming alcoholic beverages (beer, red wine, and white wine) ≥2 times a month (27). A 5 ml fasting blood sample was collected from each subject and levels of the fasting blood glucose (FBG), triglycerides (TGs), high-density lipoprotein cholesterol (HDL-C), total cholesterol (TC), low-density lipoprotein cholesterol (LDL-C), and other indicators were obtained using an automatic biochemical analyser (Olympus AU 2700; Olympus Diagnostics, Hamburg, Germany) at the First Affiliated Hospital of Shihezi University School of Medicine. In this study, individuals with diabetes (28) were defined as having FBG level of ≥7.0 mmol/L and 2-h postprandial blood glucose level of ≥11.1 mmol/L, a previous diabetes diagnosis, and use of blood sugar control drugs. We also calculated other synthetic indices, including TyG, (TG [mg/dl]^*^FBG [mg/dl]), (LAP) (men: [WC-65]^*^TC [mmol/L]; women: [WC-58]^*^TG [mmol/L]); lipoprotein combine index (LCI) (TC^*^TG [mmol/L]^*^LDL-C/HDL-C); atherogenic index (AI) (TC [mmol/L]-HDL-C)/HDL-C); atherogenic index of plasma (AIP) (Log[TG/HDL]); LpH (LDL-C/HDL-C ratio); and bilirubin comprehensive index (THT) (TC [mmol/L]/[HDL-C+TBIL (μmol/mL)]).

### Data Pre-processing

There were some missing values in the database, and direct deletion of missing values resulted in the loss of sample information. Since there were a few variables with missing values in this study, continuous variables were filled using the mean, while categorical variables were filled using the mode. By standardizing continuous variables, categorical variables were processed by one-hot encoding to reduce the influence of different variable units and quantity levels on the analysis. For the description of missing variables in this study, see Supplementary Table 1.

### Diagnostic Criteria

The diagnostic criteria for CVD (29) pertained to the detection of ischaemic heart disease, cerebrovascular disease, and related diseases [International Classification of Diseases (ICD)-9: code 390–495]; hospitalization; or death due to CVD (ICD-10) during the follow-up period. Data regarding patient questionnaire answers, medical records, and the diagnosis of CVD during the follow-up period were obtained and recorded. If the same type of CVD event occurred more than once in a patient, the first occurrence of CVD was the final event. The time of onset was recorded. Self-reported patients needed to provide proof of their clinical diagnosis.

### Introduction to Predictive Models

Logistic regression belongs to probabilistic nonlinear regression and is one of the most widely used classification models. Logistic regression usually uses regularization to optimize the model. The adjustable parameters include inverse regularization parameters and methods (30). By adding a regularization coefficient to Logistic regression, the parameters of the variable are sparse, so that the weight of most of the feature vectors is 0, thereby reducing the dimension of the variable. SVM is currently one of the most common ML algorithms that can effectively solve the classification problem of small samples and nonlinear and high-dimensional data. It classifies samples by finding a set of hyperplanes in a high-dimensional space, and the samples closest to the hyperplane are called support vectors. When the training data are inseparable, this problem can be solved using the kernel trick (31).That is, the original features of the samples are mapped to a higher dimensional space that makes the samples linearly separable through the mapping function. The RF algorithm is an ensemble learning algorithm based on the decision tree algorithm. The basic idea is to integrate weak classifiers into a more robust model (32). AdaBoost (33) is an ensemble learning algorithm based on boosting. The algorithm first builds a weak learner based on the training data and then according to AdaBoost, increases the weight of the samples that were misclassified by weak learning in the previous round. Then, it reduces the weight of the correctly classified samples, loops this process until the weak learner reaches the specified value, and then linearly combines all weak learners to obtain the final strong classifier by weighted majority voting. In this study, both random forest and Adaboost are ensemble learning algorithms based on decision trees. The decision tree algorithm selects variables by evaluating the characteristics and depth of dividing nodes, reducing the dimension of variables. The integrated model has better generalization error and can effectively reduce the overfitting combination phenomenon.

### Model Establishment and Verification

The datasets were randomly divided into training datasets (927CVD/10153) and test datasets (249CVD/2539). The KS test was performed on the training and test datasets, and the *P*-values were both >0.05. The ratio of the training and test datasets was 8:2. We considered four variable selection methods: forward partial likelihood estimation (FLR) with logistic regression (LR), lasso regularization with logistic regression (Lasso-LR), permutation-based selection with random forest (RF), and characteristic importance with RF. Variables were established using a subset of algorithms, such as L1-LR, RF, SVM, and AdaBoost. A prediction model of each algorithm was then established. The optimal prediction model of the same algorithm was then selected by discrimination and calibration, and the most suitable prediction model for the population was obtained by comparing the discrimination, calibration, and clinical effectiveness of the optimal prediction models of different algorithms.

The discrimination of the model was determined by comparing the area under the receiver operating curve (AUC), Net Reclassification Index (cNRI), and Integrated Discrimination Improvement Index (IDI) (34) between models, and the calibration degree was compared by calculating the Brier Score (BS) and Homser–Lemeshow χ^2^ (35, 36). This study evaluated the clinical validity of the model using decision curve analysis (DCA) (37). The horizontal axis of the decision curve represents the threshold probability and vertical axis represents the net benefit obtained after subtracting the harm from the benefit under the threshold probability. Using DCA to determine the net benefit that can be obtained using the model to screen high-risk groups compared with assuming that all participants are high-risk groups of CVD and implanting undifferentiated interventions, followed by calculating the net benefit without increasing the number of positive results, can reduce unnecessary interventions.

To avoid over-fitting the problem of the model in the process of model selection and hyper-parameter tuning, we used a 10-fold cross-validation to optimize the parameters of the training set and subsequently selected the optimal model. This method divided the training data in 10 equal, non-repeated parts, nine of which were used for model training, and the remaining one was used for model verification. This process was repeated 10 times, and combination of Bayesian optimisation and grid search was used to select the optimal hyperparameters. The AUC was used as the model selection criterion to determine the hyperparameter value that optimized the model predictive performance. Afterwards, we used the optimal hyperparameter value. We built the model on all training data sets. Finally, the independent test data set was used to make a final evaluation of model performance.

### Data Analysis

Since machine learning algorithms, such as SVM output, predicted CVD occurrence by default, they did not directly predict CVD probability. We used the Platt scaling method (38) to calibrate the predicted probabilities output using the four models for more accurate prediction of CVD risk and identification of high-risk groups. The data used in this study were unbalanced to enable the use of the threshold probability movement method. The default 0.5 of the model was not used as the standard for dividing the incidence of CVD. However, the optimal threshold probability of each model was determined according to the Youden Index, which was the basis for dividing the high-risk population of CVD. All statistical analyses were performed using the Python 3.7 or R version 4.0. A two-sided test with a *P*-value of <0.05 was considered statistically significant.

## Results

### Study Population Characteristics

A total of 12,692 people (6,264 men, 6,398 women; average age 41.24 years) were included in this study. A total of 1,176 CVD events were observed during a median follow-up of 4.94 years. The cumulative incidence was 9.26%. Compared with people without CVD events, those with CVD showed a higher trend in study indicators, such as age, BMI, TC, alkaline phosphatase (ALP), WC, and HC. Moreover, subjects with high blood pressure and type 2 diabetes were also at a higher risk of CVD development. The comparison of different characteristics between participants with CVD and those without training and test datasets listed is shown in Supplementary Tables 2.1,2.2.

### Independent Variable Selection and Optimal Model Construction

The research database included demographic characteristics, physical examination findings, and serology results. There were 62 variables in total. After removing the missing ratio of ≥50% and 11 variables unrelated to the research, a total of 51 variables were included. The following methods were used to filter and establish a subset of variables: FLR-LR (22 variables) and Lasso-LR (34 variables). The top 35 variables were selected according to the built-in random forest importance. The top 30 variables were subsequently selected as the screening subset according to permutation feature importance of RF. The variable subsets formed by the selected variables using the four methods are shown in Supplementary Tables 3–6.

To further explore the predictive performance of different variable subsets on different algorithms, we used the above variable subsets and the full variable set to build predictive models using different algorithms to find the algorithm based on the optimal model. Through Bayesian optimization and grid search, the hyperparameter values with the best prediction performance of each model were selected (Supplementary Tables 7.1–7.4). The AUC values of different algorithms in the training and test datasets are shown in Supplementary Table 8. There was no risk of overfitting and, to comprehensively consider the results of discrimination and calibration, this study concluded that the optimal models based on the four algorithms were Lasso-AdaBoost, FLR-L1-LR, FLR-RF, and FLR-SVM (Supplementary Tables 9.1–9.4).

### Comparison of Optimal Model Prediction Performance

**Figure 1:**
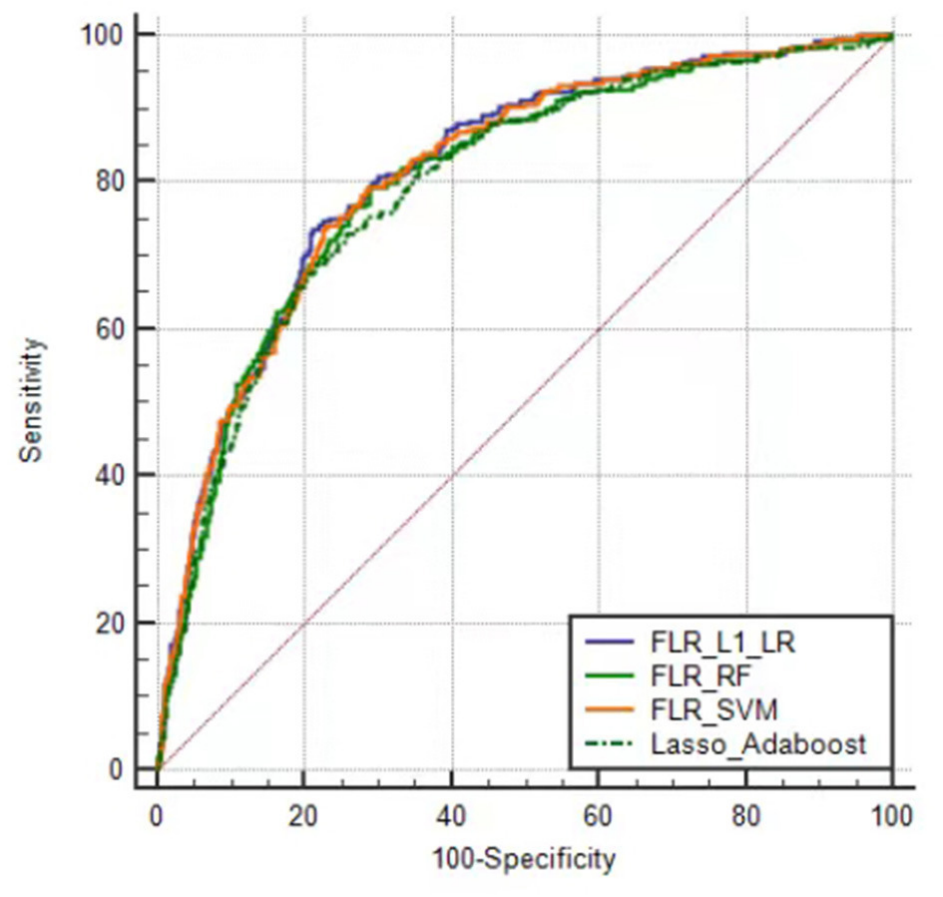
Receiver operator characteristic curves of the optimal prediction model in Xinjiang rural population. FLR-L1-LR, L1 regularized Logistic regression with forwarding Partial Likelihood Estimation; FLR-RF, Random forest with forwarding Partial Likelihood Estimation; FLR-SVM, Support vector machine with forwarding Partial Likelihood Estimation.

The predictive performance indicators of the optimal models for each algorithm are listed in Table 1. All models have a moderate or higher (AUC value between 0.798 and 0.817) distinguishing ability. The AUC of FLR-L1-LR, FLR-SVM, FLR-RF, and Lasso-AdaBoost was 0.817 (95% CI, 0.801–0.832), 0.814 (95% CI, 0.798–0.829), 0.804 (95% CI, 0.788–0.820), and 0.798 (95% CI, 0.782–0.81), respectively. The receiver operating characteristic (ROC) curve of the prediction model is shown in Figure 1.

**Table 1:** Comparison of the prediction performance of the optimal model of each algorithm.

**Model**	**AUC**	**Youden** **Index**	**Optimal** **threshold**	**Sensitivity** **(%)**	**Specificity** **(%)**	**PPV (%)**	**NPV (%)**	**Proportion of** **high-risk** **population (%)**	**Brier score**	**Homser-** **Lemeshow** **χ^**2**^**	***P-*Value**
Lasso-AdaBoost	0.798 (0.782, 0.813)	0.472	0.11	73.09	74.10	23.5	96.2	30.4	0.078 (0.070, 0.086)	13.81	0.09
FLR-L1-LR	0.817 (0.801, 0.832)	0.524	0.11	73.49	78.86	27.4	96.5	26.7	0.076 (0.069, 0.084)	11.51	0.17
FLR-RF	0.804 (0.788, 0.820)	0.506	0.08	79.52	71.09	23.0	97.0	33.1	0.077 (0.070, 0.086)	11.59	0.17
FLR-SVM	0.814 (0.798, 0.829)	0.511	0.11	73.90	77.16	26.0	96.5	38.4	0.076 (0.069, 0.084)	16.10	0.04

*AUC, area under the receiver operating characteristic curve; PPV, positive predictive value; NPV, negative predictive value; Lasso-AdaBoost, AdaBoost with Lasso regression; FLR-L1-LR, L1 regularized Logistic regression with forward Partial Likelihood Estimation; FLR-RF, random forest with forward Partial Likelihood Estimation; FLR-SVM, support vector machine with forward Partial Likelihood Estimation*.

**Figure 2:**
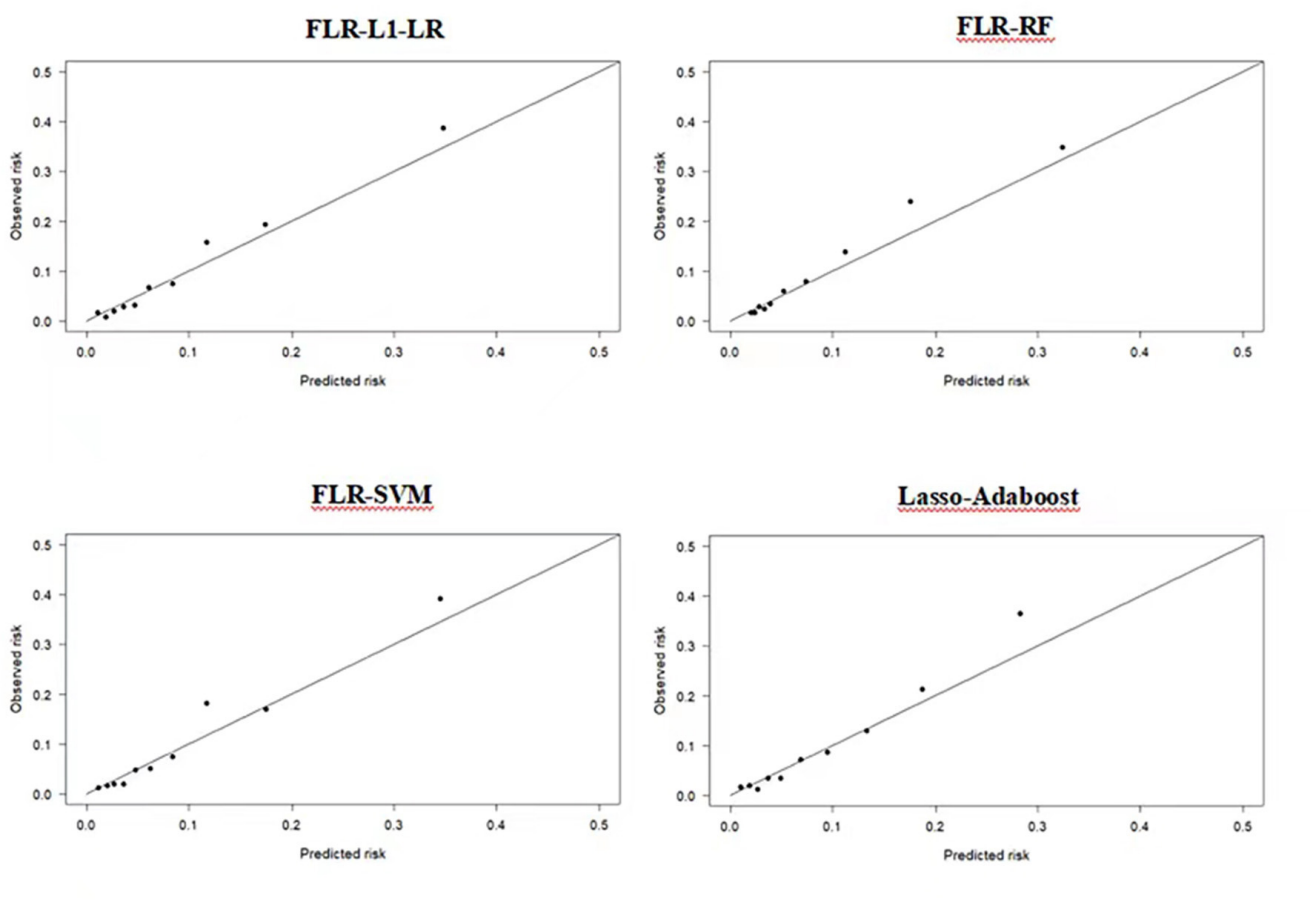
Calibration plots of four ML models in predicting CVD outcomes in Xinjiang rural population. CVD, cardiovascular disease; ML, machine learning; FLR-L1-LR, L1 regularized Logistic regression with forwarding Partial Likelihood Estimation; FLR-RF, Random forest with forwarding Partial Likelihood Estimation; FLR-SVM, Support vector machine with forwarding Partial Likelihood Estimation.

Compared with other optimal models, the FLR-L1-LR model performed better in terms of Youden index, specificity, and PPV when the optimal threshold was 0.11. BS and Homser–Lemeshow χ^2^ also demonstrated that the FLR-L1-LR model was better than others. In the FLR-L1-LR model, 26.7% of the participants were identified as high risk for CVD development (Table 1). The results of the calibration curve showed that FLR-L1-LR, FLR-SVM, Lasso-AdaBoost, and FLR-RF predicted the number of patients with CVD to be 234.12, 234.05, 230.55, and 223.93, respectively. The corresponding predicted CVD events/objective CVD events (P/O) values were 94.02, 94.00, 92.59, and 89.93, respectively (Figure 2).

To further select a prediction model suitable for this population, we compared the differences between the AUC value, IDI, and cNRI of the optimal models. We found that the AUC values of FLR-L1-LR and FLR-SVM were similar (*P* > 0.05), and both were higher than the AUC values of Lasso-AdaBoost and FLR-RF (*P* < 0.05). The reclassification capabilities of each model were compared with that of the FLR-L1-L model. The cNRI values of FLR-SVM and Lasso-AdaBoost values were 0.278 and 0.208, respectively. Compared with the FLR-L1-LR model, the Lasso-AdaBoost and the FLR-SVM models had a correct classification rate of 21 and 28%, respectively. Similarly, FLR-SVM was compared with Lasso-AdaBoost in terms of the proportion of correct classification. The FLR-SVM had a 17% increased proportion of correct classification compared with that of the Lasso-AdaBoost. The difference between the reclassification capabilities of the remaining models was not statistically significant. The results of the comprehensive discrimination ability of each model, from best to worst, were FLR-L1-LR > FLR-SVM > FLR-RF > Lasso-AdaBoost. This is described in Table 2.

**Table 2:** Comparison of discrimination performance of optimal prediction models.

**Predictive model**	**AUC difference**	***P*-Value**	**cNRI**	***P*-Value**	**IDI**	***P*-Value**
Lasso-AdaBoost vs. FLR-L1-LR	0.019	0.002	0.208 (0.078, 0.337)	<0.001	0.032 (0.019, 0.045)	<0.010
Lasso-AdaBoost vs. FLR-RF	0.007	0.334	0.097 (−0.033, 0.228)	0.143	0.016 (0.007, 0.025)	<0.010
Lasso-AdaBoost vs. FLR-SVM	0.016	0.047	0.167 (0.037, 0.296)	0.012	0.029 (0.016, 0.042)	<0.010
FLR-RF vs. FLR-L1-LR	0.012	0.045	0.108 (−0.022, 0.238)	0.105	0.016 (0.003, 0.028)	0.010
FLR-RF vs. FLR-SVM	0.003	0.016	0.072 (−0.058, 0.203)	0.278	0.013 (0.001, 0.026)	0.040
FLR-SVM vs. FLR-L1-LR	0.010	0.118	0.278 (0.149, 0.408)	<0.001	0.003 (0.001, 0.004)	<0.010

*AUC, area under the receiver operating characteristic curve; cNRI, continuous Net Reclassification Index; IDI, Integrated Discrimination Improvement Index; Lasso-AdaBoost, AdaBoost with Lasso regression; FLR-L1-LR, L1 regularized Logistic regression with forward Partial Likelihood Estimation; FLR-RF, random forest with forward Partial Likelihood Estimation; FLR-SVM, support vector machine with forward Partial Likelihood Estimation*.

**Figure 3:**
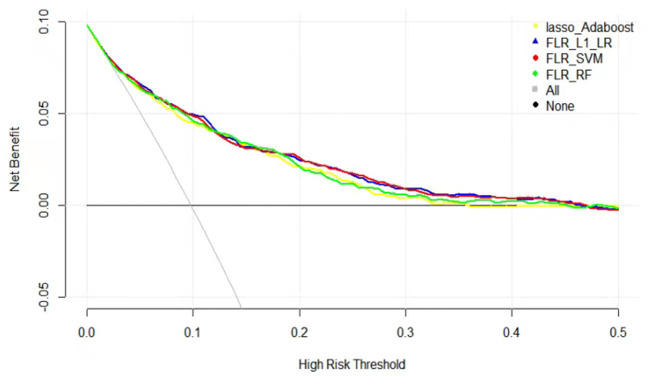
Decision curves for predicting CVD outcomes in Xinjiang rural population using four ML models. CVD, cardiovascular disease; ML, machine learning; FLR-L1-LR, L1 regularized Logistic regression with forwarding Partial Likelihood Estimation; FLR-RF, Random forest with forwarding Partial Likelihood Estimation; FLR-SVM, Support vector machine with forwarding Partial Likelihood Estimation.

The clinical effectiveness of FLR-L1-LR, FLR-SVM, FLR-RF, and Lasso-AdaBoost based on the results of the decision curve are shown in Figure 3. It is evident that the clinical application value of the FLR-L1-LR model is higher than that of FLR-SVM, Lasso-AdaBoost, and FLR-RF (Figure 3, Table 3). Under the optimal threshold, we assumed that all participants were in a high-risk group for CVD. We then administered undifferentiated interventions for primary and secondary prevention. The net benefit of using the FLR-L1-LR model was 0.061. This showed that without increasing the positive results, 49 out of every 1,000 people could avoid unnecessary interventions.

**Table 3:** Comparison of clinical effectiveness of models.

**Model**	**Pt (%)**	**Net income**		**Model** **net** **income**	**Advantages** **of the** **model**^**#**^
		**Treat all**	**Prediction** **model**		
FLR-L1-LR	5	0.051	0.066	0.015	29
	10	−0.002	0.049	0.051	46
	11^a^	−0.013	0.048	0.061	49
FLR-SVM	5	0.051	0.065	0.014	27
	10	−0.002	0.048	0.050	45
	11^a^	−0.013	0.045	0.058	47
Lasso- AdaBoost	5	0.051	0.063	0.012	23
	10	−0.002	0.045	0.047	43
	11^a^	−0.013	0.043	0.056	46
FLR-RF	5	0.051	0.064	0.013	25
	10	−0.002	0.046	0.048	43
	8^a^	0.02	0.053	0.033	38

# *The value was calculated as: (net benefit of the model– net benefit of treat all)/[pt/(1 – pt)] × 100.*

a *Select the optimal threshold probability of each model according to AUC.*

*Pt, Threshold probability; Lasso-AdaBoost, AdaBoost with Lasso regression; FLR-L1-LR, L1 regularized Logistic regression with forward Partial Likelihood Estimation; FLR-RF, random forest with forward Partial Likelihood Estimation; FLR-SVM, support vector machine with forward Partial Likelihood Estimation*.

### Variable Importance Ranking of the Optimal Model Output

**Figure 4:**
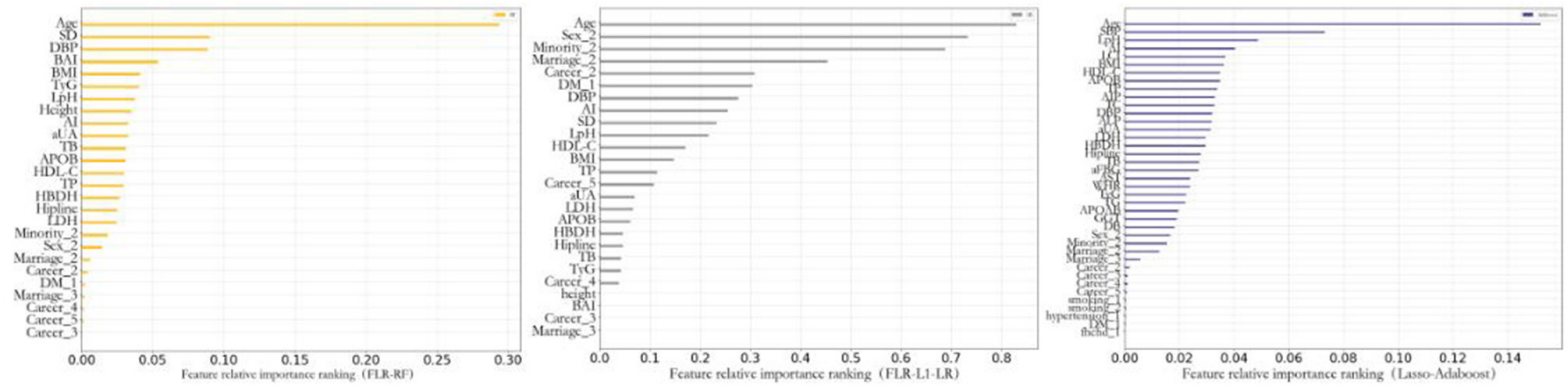
Feature importance of included variables obtained from the random forest with forwarding Partial Likelihood Estimation (FLR-RF), L1 regularized Logistic regression with FLR (FLR-L1-LR), Lasso-AdaBoost model. SD, pulse pressure difference; DBP, diastolic blood pressure; BAI, body obesity index; BMI, body mass index; TyG, triglyceride blood glucose index; LpH, low-high-density lipoprotein ratio; AI, arteriosclerosis index; aUA, uric acid; TB, total bilirubin; APOB, apolipoprotein B; HDL-C, high-density lipoprotein cholesterol; TP, total protein; HBDH, α-hydroxybutyrate dehydrogenase; LDH, lactate dehydrogenase; SBP, systolic blood pressure; LCI, blood lipid index; AIP, Plasma arteriosclerosis index; TC, total cholesterol; ALP, alkaline phosphatase; aFBG, fasting blood glucose; AST, aspartate aminotransferase; WHR, waist-to-height ratio; APOAB, apolipoprotein AB; GGT, γ-glutaminase; DB, Direct Bilirubin; DM, diabetes mellitus; Fhchd, Family history of coronary heart disease.

Previous studies indicated that compared with FRS and PCE, the ML algorithm could better determine the nonlinear and complex relationships between variables and outcomes. Furthermore, the ML algorithm identified potential risk factors more effectively (39–41). We further analyzed the relative relationship among the importance rankings of the algorithm variables using the coefficients of variables that could not be obtained based on the Gaussian kernel function. Therefore, this study only highlights the importance of the optimal model variables established by the AdaBoost, RF, and L1-LR algorithms to compare the ability of each variable to predict the incidence of CVD (Figure 4). This study found that the risk factors for CVD included factors that reflected the degree and type of body obesity, such as age, sex, ethnicity, DBP, HDL-C level, TC level, BAI, and BMI. Risk factors also included those that reflected glucose and lipid metabolism, such as TyG, LpH level, AI, and occupation type. The indicators were also risk factors for CVD and could predict CVD risk.

## Discussion

The results of this study show that the cumulative incidence of CVD in the Xinjiang Uyghur and Kazak populations was 9.26%. The incidence was similar to that in African Americans (42). However, it was higher than those of Han Chinese ancestry (43–45), which may relate to the population's unique genetic background and diet. Here, we used ML algorithms to establish a predictive model and discover the main factors for the occurrence of CVD in this population.

To achieve the best predictive performance of the established model, we selected variables through four variable screening methods. We subsequently established different variable subsets, unlike those in the previous study that only used the feature importance of the RF algorithm to select variables (46). Our results indicate that the subset of variables established using FLR showed the best performance on the L1-LR, RF, and SVM algorithms, similar to the results reported by De Silva et al. (47). Unlike other variable screening methods, FLR focused more on the linear relationship between variables. The model built based on the combination of FLR-screened variable subsets and other ML algorithms had better predictive performance. This may be due to the consideration of the linear relationship of variables based on logistic regression and the in-depth analysis of the nonlinear relationship using different machine learning algorithms.

When the optimal prediction models of the LR, SVM, RF, and AdaBoost algorithms were compared, the prediction performance of the LR-based model was better than that of the other ML algorithm models. These findings are similar to those of a 2019 systematic review (15). There are many possible reasons for this phenomenon. First, the number of variables included in this research was limited, and some ML algorithms were better at dealing with high-dimensional data problems. Moreover, the logistic regression model was established based on the L1 regularization method. This method was better at dealing with small samples and low-dimensional data and was not easily affected by outliers. The established model was more robust.

Second, the performance of the SVM-based prediction model was lower than that of LR but higher than those of RF and AdaBoost. These findings are similar to the those reported by Wallert et al. (48). This might be because, although the SVM model based on the Gaussian kernel function could handle the nonlinear relationship among variables well, when dealing with research with fewer variables, its prediction performance was affected by insufficient variables. Prediction performance was lower in the SVM model compared with that of LR. Due to the poor interpretability of SVM and the difficulty of parameter optimisation, the model has fewer clinical application. Nevertheless, its high predictive potential was not ruled out.

Finally, concerning the RF and AdaBoost algorithms, the prediction performance of RF in this study was better than that of AdaBoost, although both integrated learning algorithms. Nevertheless, both were lower than those of LR and SVM, which are consistent with the results of Hae et al. (49). This may be because, compared with a single algorithm, integrated learning algorithms such as RF and AdaBoost require a larger sample size to achieve the optimal model performance (50). Therefore, it did not show optimal performance with the medium sample size of this study.

A comprehensive analysis of the variable importance rankings of the three algorithms revealed that age and systolic blood pressure were the most important predictors. This was similar to the findings of previous studies (9, 51). Furthermore, this study found that compared with a single blood lipid index, composite indicators such as LpH and TyG calculated from multiple blood lipid indicators showed better predictive performance. Similarly, in a study by Huang et al. (52), compared with HDL-C and LDL-C alone, LpH had a stronger correlation with the severity of coronary heart disease. The results of the Tehran Lipid and Glucose Metabolism Study showed that for every standard deviation increase of 1 in TyG, the individual CVD risk increased by 20% (53). In addition, similar studies showed that TyG was an important variable of CVD risk prediction. This was similar to the results of this study (54). BMI and BAI were indicators that reflected the degree and type of body obesity. Moreover, related research showed that it had value in predicting CVD incidence (55, 56). The results of this study also showed that BMI and BAI had strong capabilities of CVD prediction. This may be due to the high-salt and high-fat diets of the Uyghur and Kazakh populations, resulting in high body weight and large hip circumference.

Although we believe that the included population represents the general Uyghur and Kazak populations, this study has certain limitations. First, the variable information included was relatively small. ML algorithms are good at dealing with data relationships between high-dimensional data. The reduced sample information in this study may be the main reason for the limited prediction performance of ML algorithms. Second, this study lacked an independent external verification population, and the prediction accuracy and robustness of extrapolating the established model to other ethnic populations needs to be explored further. Moreover, only the baseline measurement data were used for modeling. Time effect and censored data were not considered during model construction. Finally, although this study uses Plating scaling to deal with this imbalanced dataset, the positive predictive value of different models in this population is low, which may lead to unnecessary intervention in the population.

## Conclusion

In this study, the performance of the CVD prediction model based on the L1-LR algorithm was higher than those of other ML algorithms. In addition to the traditional single risk factors for cardiovascular disease, complex lipid metabolism indicators, such as LpH and TyG, and obesity indicators, such as BMI and BAI, were found to be important factors for predicting the incidence of CVD in this population.

## Data Availability Statement

The raw data supporting the conclusions of this article will be made available by the authors, without undue reservation.

## Ethics Statement

The studies involving human participants were reviewed and approved by Ethics Committee of the First Affiliated Hospital of Shihezi University School of Medicine (No. SHZ2010LL01). The patients/participants provided their written informed consent to participate in this study.

## Author Contributions

XQ and YL designed the study, analyzed the data, and wrote the manuscript. XHZ, HG, and JH collected and sorted the data. XPW, YZY, and JLM sorted and checked the data. SXG and RLM designed the study, guided the article writing, and modified the manuscript. All authors contributed to the article and approved the submitted version.

## Funding

This research was funded by the Non-profit Central Research Institute Fund of Chinese Academy of Medical Sciences (2020-PT330-003), the Shihezi University Innovation Outstanding Young Talents Program (Natural Science) (No. CXPY202004), and Shihezi University independently funded and supported school-level scientific research projects (No. ZZZC202018A).

## Conflict of Interest

The authors declare that the research was conducted in the absence of any commercial or financial relationships that could be construed as a potential conflict of interest.

## Publisher's Note

All claims expressed in this article are solely those of the authors and do not necessarily represent those of their affiliated organizations, or those of the publisher, the editors and the reviewers. Any product that may be evaluated in this article, or claim that may be made by its manufacturer, is not guaranteed or endorsed by the publisher.

## Abbreviations

CVD: cardiovascular disease
ML: machine learning
L1-LR: L1 regularized logistic regression
RF: random forest
SVM: support vector machine
SBP: systolic blood pressure
TyG: triglyceride blood glucose index
BMI: body mass index
BAI: body obesity index
TG: triglycerides
HDL-C: high-density lipoprotein cholesterol
DBP: diastolic blood pressure
WHR: waist-to-hip ratio
LCI: lipoprotein combine index
AI: atherogenic index
LpH: low-high-density lipoprotein ratio
THT: bilirubin comprehensive index
FLR: forward partial likelihood estimation
LR: logistic regression
RF: Random forest
AUC: the area under the receiver operating curve
cNRI: the Net Reclassification Index
IDI: Integrated Discrimination Improvement Index
BS: Brier Score.
