# Supplementary material for: A Cardiovascular Disease Prediction Model Based on Routine Physical Examination Indicators Using Machine Learning Methods: A Cohort Study
Source: Front Cardiovasc Med. 2022 Jun 17;9:854287. doi: 10.3389/fcvm.2022.854287 (PMC9247206; doi:10.3389/fcvm.2022.854287)
Supplement: Supplementary file 1 [file Data_Sheet_1.pdf]

10.3389/fcvm.2022.854287

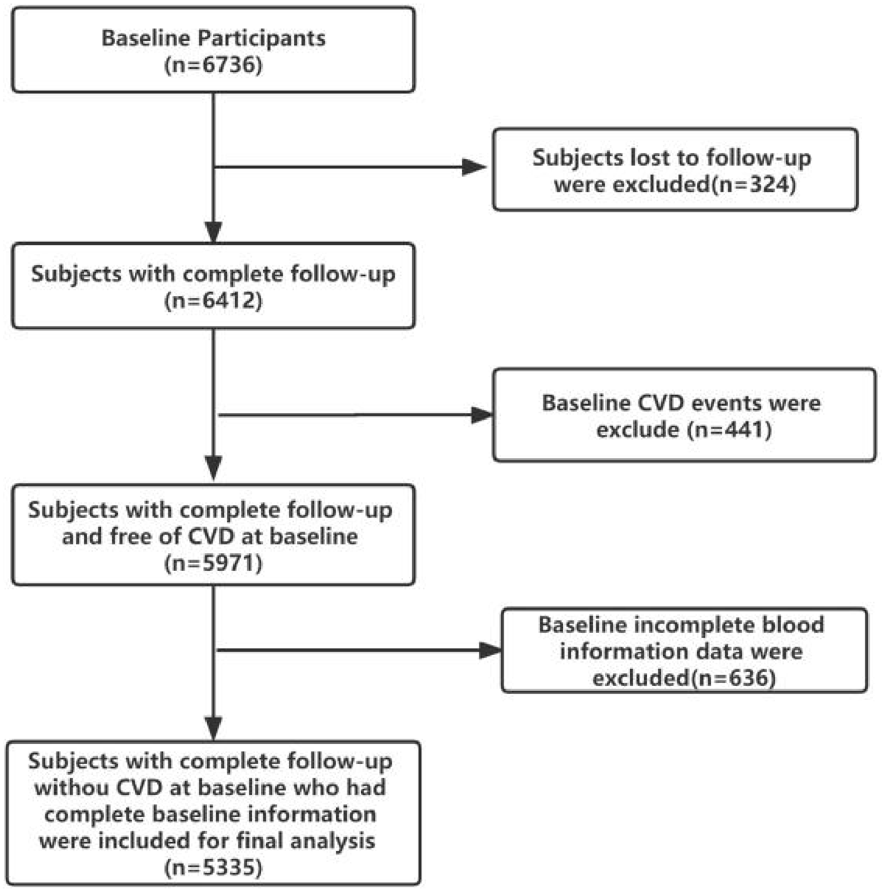

Supplementary: Fig. 1.1 Flow diagram of subjects included of cohort 1.CVD, Cardiovascular Disease.

10.3389/fcvm.2022.854287

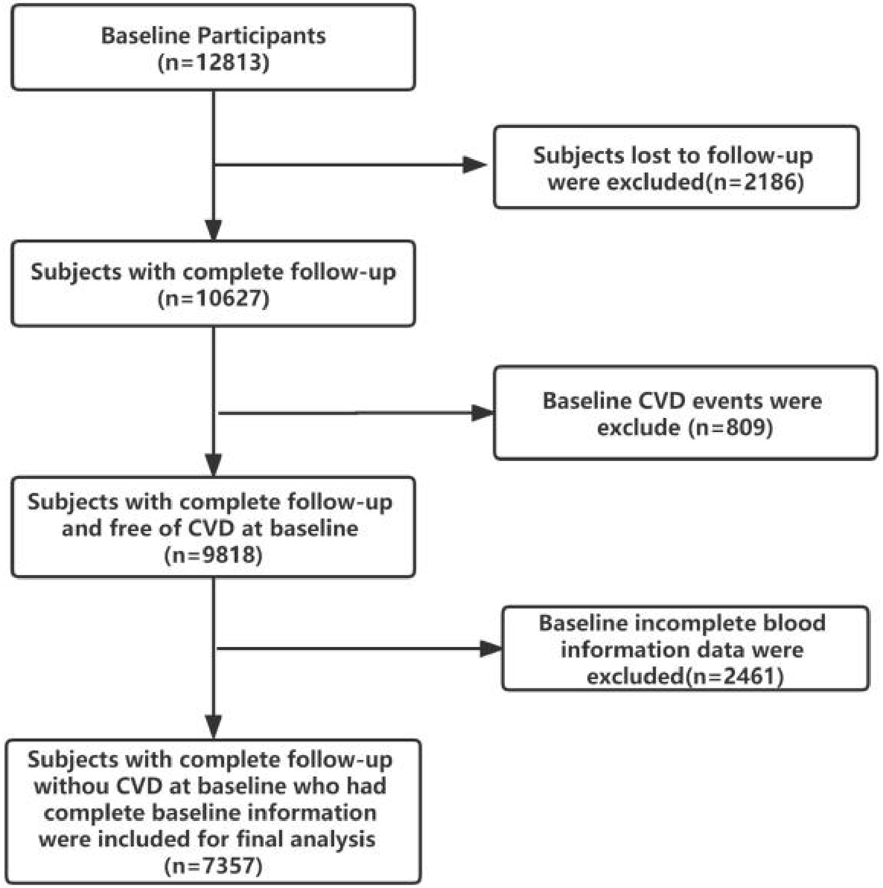

Supplementary: Fig. 1.2 Flow diagram of subjects included of cohort 2.CVD, Cardiovascular Disease.

10.3389/fcvm.2022.854287

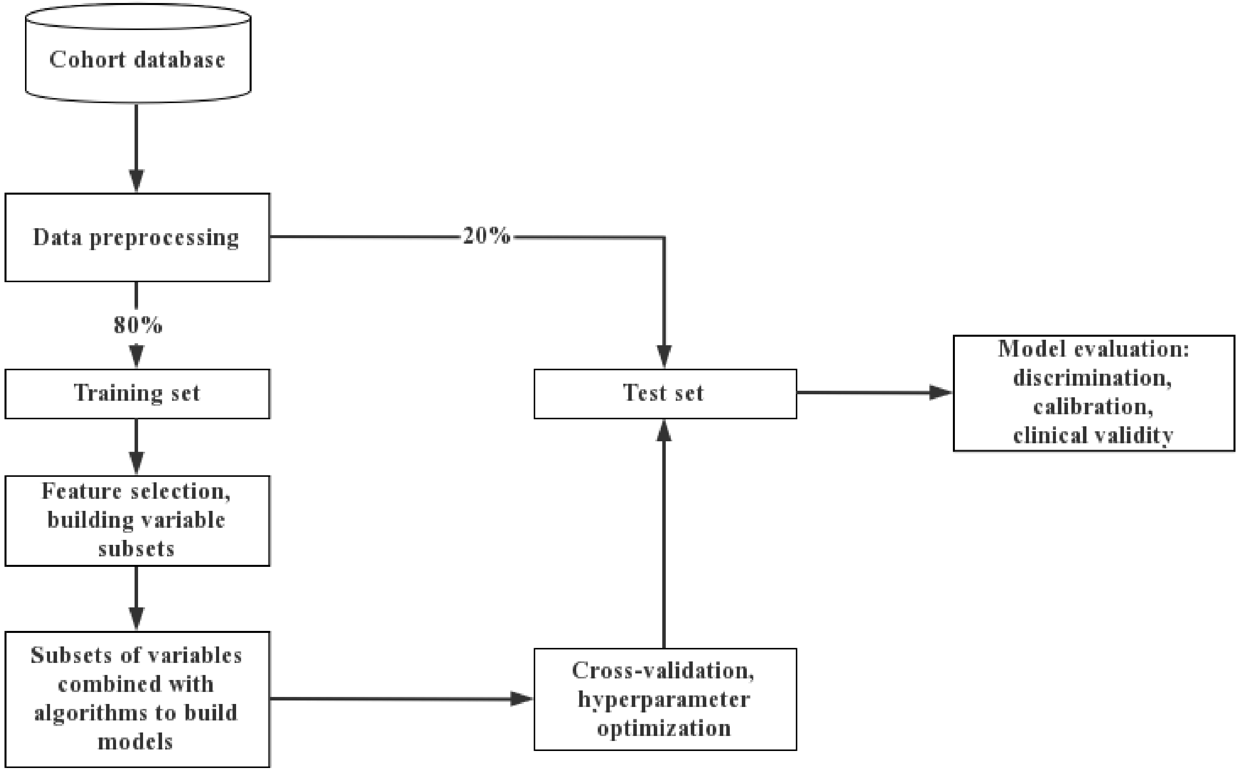

Supplementary: Fig. 1.3 Flow diagram of the data analysis process in this study

The research database included demographic characteristics, physical examination findings, and serology results. There were 62 variables in total. After removing the missing ratio of ≥50% and 11 variables unrelated to the research, a total of 51 variables were included. Since there were a few variables with missing values Included in the analysis in this study, continuous variables were filled using the mean, while categorical variables were filled using the mode.

Supplementary: Table 1 Missing variable descriptions

Variable	Missing ratio	Variable	Missing ratio
Education level	1.06%	Self-assessment of the health status of the elderly	70.00%*
Profession	2.00%	Self-assessment of self-care ability of the elderly	70.00%*
Waist circumference	1.19%	Self-assessment of cognitive function in the elderly	70.00%*
Hip circumference	1.07%	Self-assessment of emotional state of the elderly	70.00%*
Height	3.34%	Type of drinking water	65.00%*
Weight	2.78%	Fuel type	65.00%*
Exercise frequency	67.30%*	Exercise type	67.30%*
Each exercise time	67.30%*	Exercise frequency	67.30%*
Stick to exercise time	67.30%*	Kitchen exhaust facility	51.00%*

Note:*Missing values greater than 50% were not included in the analysis

Divide the data set into training set and test set according to 2:8, compare whether there is a difference between the count data between the CVD group and the non-CVD group on the training set and the test set. Two-sided test *P<0.05*.

Supplementary: Table 2.1 Comparison of research objects between training set and test set (counting data)

Feature	Training set			Test set		
	Non-CVD（9226）	CVD（927）	*P* value	Non-CVD (2290)	CVD (249)	*P value*
Age(years)	40.04±13.53	53.22±12.86	<0.01	39.96±13.74	53.18±13.40	<0.01
Height(cm)	162.51±8.61	159.85±8.10	<0.01	162.49±8.54	159.41±8.46	<0.01
Weight(Kg)	66.01±13.11	68.51±14.46	<0.01	65.84±13.33	68.04±15.77	0.01
WC(cm)	88.65±12.51	93.58±13.56	<0.01	88.63±12.86	93.35±14.01	<0.01
HC(cm)	98.85±8.22	101.22±9.48	<0.01	98.78±8.35	100.49±10.18	<0.01
FBG (mmol/L)	5.00±1.55	5.54±2.58	<0.01	4.96±1.45	5.43±2.09	<0.01
TG (mmol/L)	1.56±1.28	1.76±1.47	<0.01	1.57±1.38	1.84±1.48	<0.01
TC (mmol/L)	4.56±1.80	4.72±1.15	<0.01	4.53±2.01	4.85±1.42	0.01
HDL-C(mmol/L)	1.43±0.56	1.36±0.48	<0.01	1.43±0.63	1.37±0.46	0.11
LDL-C(mmol/L)	2.46±0.89	2.64±0.77	<0.01	2.43±0.79	2.68±0.89	<0.01
SBP (mmHg)	127.13±19.63	145.13±26.88	<0.01	126.58±12.42	145.66±27.10	<0.01
DBP (mmHg)	76.80±12.71	85.07±15.42	<0.01	76.59±12.42	85.14±15.77	<0.01
ALT (mmol/L)	24.46±22.23	23.30±20.53	0.13	24.47±21.32	21.84±14.24	0.06
AST (mmol/L)	24.80±15.41	25.73±13.46	0.08	24.83±16.68	25.18±10.61	0.74

TP (mmol/L)	74.02±9.24	73.52±11.25	0.13	73.70±9.76	74.07±11.04	0.58
ALB(g/L)	76.75±76.54	96.64±96.70	<0.01	75.95±75.32	100.23±98.03	<0.01
GLO(g/L)	29.77±5.05	30.74±5.56	<0.01	29.65±5.26	30.76±5.26	<0.01
TB(umol/L)	10.53±6.09	10.74±7.00	0.32	10.31±6.11	10.38±5.82	0.85
DB(umol/L)	3.78±2.47	3.63±2.11	0.06	3.67±2.30	3.76±4.12	0.60
ALP(U/L)	78.31±30.76	81.22±26.77	<0.01	77.78±28.23	85.34±32.36	<0.01
GGT(U/L)	19.22±19.92	20.76±15.83	0.02	18.67±15.38	23.38±24.67	<0.01
CR(umol/L)	66.43±16.50	66.64±20.37	0.71	66.35±16.92	66.01±17.22	0.77
AOPAB(g/L)	1.39±0.85	1.34±1.11	<0.01	1.39±0.61	1.33±0.55	0.10
AOPB(g/L)	0.92±0.85	1.34±1.10	<0.01	0.91±0.26	0.98±0.31	<0.01
HBDH(U/L)	146.31±56.84	150.08±61.11	0.06	146.08±55.11	153.16±84.50	0.07
LDH(U/L)	173.31±59.47	179.67±68.04	<0.01	173.94±59.03	188.49±102.52	<0.01
SD(mmHg)	50.32±14.83	60.07±19.90	<0.01	49.83±14.54	60.52±18.46	<0.01
WHR	0.89±0.09	0.92±0.10	<0.01	0.89±0.09	0.93±0.11	<0.01
BMI(Kg/m^2)	24.95±4.40	26.78±5.22	<0.01	24.90±4.53	26.65±5.37	<0.01
aUA(umol/L)	284.60±87.44	294.59±117.64	<0.01	284.65±85.74	297.59±94.56	0.03
TyG	4.69±4.57	5.64±7.41	<0.01	4.64±3.81	5.70±5.46	<0.01
LAP	72.35±75.88	82.17±80.62	<0.01	71.24±77.43	85.83±87.65	<0.01
BAI	29.91±5.09	32.30±5.92	<0.01	29.88±5.06	32.07±5.56	<0.01
LCI	16.16±49.85	19.98±24.60	0.02	15.55±21.38	22.30±27.17	<0.01
AI	2.51±2.85	2.81±2.72	<0.01	2.48±2.09	2.81±1.32	0.02

AIP	-0.03±0.33	0.05±0.31	<0.01	-0.03±0.32	0.05±0.35	<0.01
LpH	1.92±1.36	2.18±1.82	<0.01	1.90±1.00	2.14±0.92	<0.01
THT	0.70±0.50	0.73±0.49	0.04	0.71±0.57	0.75±0.53	0.04

Abbreviations: continuous variables are expressed as mean ± standard deviation, CVD, cardiovascular disease; WC, waist circumference; HC,hip circumference;FBG, fasting blood glucose; TG, triglycerides; TC, total cholesterol; HDL-C, high-density lipoprotein cholesterol; LDL-C, low-density lipoprotein cholesterol; SBP, systolic blood pressure; DBP, diastolic blood pressure; ALT, alanine aminotransferase; AST, aspartate aminotransferase; TP, total protein; ALB, albumin; GLO, globulin; TB, total bilirubin; DB, direct bilirubin; ALP, alkaline phosphatase; GGT, γ-glutaminase; CR, creatinine; APOAB, apolipoprotein AB; APOB apolipoprotein B; HBDH, α-hydroxybutyrate dehydrogenase; LDH, lactate dehydrogenase; SD, pulse pressure difference ; WHR, waist-to-height ratio, BMI, body mass index; aUA, uric acid; TyG, triglyceride blood glucose index; LAP, fat accumulation product index; BAI, body obesity index; LCI, blood lipid index; AI, arteriosclerosis index; AIP, plasma arteriosclerosis index; LpH, low-high-density lipoprotein ratio; THT, bilirubin comprehensive index.

Divide the data set into training set and test set according to 2:8, compare whether there is a difference between the count data between the CVD group and the non-CVD group on the training set and the test set. Two-sided test *P<0.05*.

Supplementary: Table 2.2 Comparison of research objects between training set and test set (measurement data)

Feature		Training set			Test set		
		Non-CVD (9226)	CVD (927)	*P* value	Non-CVD (2290)	CVD (249)	*P* value
FHDM,n(%)	No	8,934(96.8)	910(98.2)	0.03	2,228(97.2)	241(96.8)	0.64
	Yes	292(3.2)	17(1.8)		62(2.8)	8(3.2)	

Fhstroke,n(%)	No	8,904(96.5)	897(96.8)	0.69	2,205(96.3)	240(96.4)	0.93
	Yes	322(3.5)	30(3.2)		85(3.7)	9(3.6)	
Fhchd,n(%)	No	8,969(97.2)	893(96.3)	0.13	2,232(94.5)	242(97.2)	0.79
	Yes	257(2.8)	34(3.7)		58(5.5)	7(2.8)	
Dm,n(%)	No	8,837(95.8)	832(89.8)	<0.01	2,193(95.8)	221(88.8)	<0.01
	Yes	389(4.2)	95(10.2)		97(4.2)	28(11.2)	
Hypertension,n(%)	No	6,159(66.8)	321(34.6)	<0.01	1,559(68.1)	91(36.5)	<0.01
	Yes	3,067(33.2)	606(65.4)		731(31.9)	158(63.5)	
Sex,n(%)	Male	4,708(51.0)	352(38.0)	<0.01	1,149(50.2)	85(34.1)	<0.01
	Female	4,518(49.0)	575(62.0)		1,141(49.8)	164(65.9)	
Nationality,n(%)	Kazakh	1,577(17.1)	251(27.1)	<0.01	385(16.8)	72(28.9)	<0.01
	Uyghr	7,649(82.9)	676(72.9)		1,905(83.2)	177(71.1)	
Profession,n(%)	Agriculture	5,007(54.3)	521(56.2)	<0.01	1,208(52.8)	125(50.2)	<0.01
	Animal husbandry	2,577(27.9)	214(23.1)		675(29.5)	59(23.7)	
	General staff	411(4.4)	29(3.1)		109(4.8)	15(6.1)	
	Housework or retirement	468(5.1)	83(9.0)		119(5.2)	25(10.0)	
	Other	763(8.3)	80(8.6)		179(7.7)	25(10.0)	
Education level,n(%)	Illiteracy	2,681(29.1)	367(39.6)	<0.01	640(27.9)	89(35.7)	<0.01
	Primary school	3,212(34.8)	365(39.4)		835(36.5)	100(40.2)	
	Junior high school	2,619(28.3)	145(15.6)		636(27.7)	37(14.9)	
	High school	457(5.0)	36(3.9)		102(4.5)	10(4.0)	

	College degree and above	257(2.8)	14(1.5)		77(3.4)	13(5.2)	
Marital status,n(%)	Married	7,984(86.5)	795(85.8)	<0.01	2,013(87.9)	200(80.3)	<0.01
	Unmarried	654(7.1)	13(1.4)		153(6.7)	5(2.0)	
	Live alone	588(6.4)	119(12.8)		124(5.4)	44(17.7)	
Smoking,n(%)	Not smoke	7,453(80.8)	765(82.5)	0.20	1,863(81.4)	204(81.9)	0.83
	Smoking	1,773(19.2)	162(17.5)		427(18.6)	45(18.1)	
Drinking,n(%)	No alcohol	8,621(93.4)	897(96.8)	0.02	2,193(95.8)	239(96.0)	0.87
	Drinking	605(6.6)	30(3.2)		97(4.2)	10(4.0)	
BPcate,n(%)	Normal blood pressure	4,472(48.5)	217(23.4)	<0.01	1,155(50.5)	60(24.1)	<0.01
	Prehypertension	2,253(24.4)	171(18.4)		566(24.7)	42(16.9)	
	Hypertension	2,501(27.1)	539(58.2)		569(24.8)	147(59.0)	

Abbreviations:Measurement data are expressed as n(%); FHDM,Family history of diabetes; Fhstroke,Family history of stroke; Fhchd,Family history of coronary heart disease;Dm, diabetes mellitus; BPcate,Blood Pressure classification.

Supplementary: Table 3 Screening variable subsets based on Logistic regression forward partial likelihood estimation method

Variable	Variable type	Variable	Variable type
DM	Classification	APOB	Continuous
Minority	Classification	HBDH	Continuous
Sex	Classification	LDH	Continuous
Career	Classification	SD	Continuous
Marriage	Classification	BMI	Continuous
Age	Continuous	aUA	Continuous
Height	Continuous	TyG	Continuous
Hipline	Continuous	BAI	Continuous
HDL-C	Continuous	AI	Continuous
DBP	Continuous	LpH	Continuous
TP	Continuous	TB	Continuous

Abbreviations:DM,diabetes mellitus; HDL-C,high-density lipoprotein cholesterol; DBP,diastolic blood pressure; TP, total protein; APOB apolipoprotein B; HBDH, α-hydroxybutyrate dehydrogenase; LDH, lactate dehydrogenase; SD, pulse pressure difference; BMI, body mass index; aUA, uric acid; TyG, triglyceride blood glucose index; BAI, body obesity index; AI, arteriosclerosis index; LpH, low-high-density lipoprotein ratio; TB, total bilirubin.

Based on logistic regression algorithm forward biased likelihood estimation method (FLR) and Lasso regression and Random forest (RF) feature importance (CI), permutation importance (PI) method to screen variables, and the selected variables Create a subset of variables, see Table 3-6.

Supplementary: Table 4 Filter subsets of variables based on Lasso-Logistic regression

Variable	Variable type	Variable	Variable type
Age	Continuous	BMI	Continuous
Sex	Classification	HDL-C	Continuous
Minority	Classification	APOB	Continuous
Marriage	Classification	TP	Continuous
SBP	Classification	AIP	Continuous
Career	Classification	TC	Continuous
LpH	Continuous	Smoking	Classification
DM	Classification	DB	Continuous
fhchd	Classification	Hipline	Continuous
AI	Continuous	TB	Continuous
LCI	Continuous	Hypertension	Classification
FBG	Continuous	WHR	Continuous
AST	Continuous	TyG	Continuous
APOAB	Continuous	TG	Continuous
DBP	Continuous	aUA	Continuous
ALP	Continuous	LDH	Continuous

10.3389/fcvm.2022.854287

HBDH	Continuous	GGT	Continuous

Abbreviations:SBP, systolic blood pressure; LpH, low-high-density lipoprotein ratio;DM,diabetes mellitus; Fhchd, Family history of coronary heart disease; AI, arteriosclerosis index; LCI, blood lipid index; FBG, fasting blood glucose; AST, aspartate aminotransferase; APOAB, apolipoprotein AB; DBP, diastolic blood pressure; ALP, alkaline phosphatase; HBDH, α-hydroxybutyrate dehydrogenase; BMI, body mass index; HDL-C, high-density lipoprotein cholesterol; APOB apolipoprotein B; TP, total protein; AIP, Plasma arteriosclerosis index; TC, total cholesterol; DB, Direct Bilirubin; TB, total bilirubin; WHR, waist-to-height ratio; TyG, triglyceride blood glucose index;aUA, uric acid; LDH, lactate dehydrogenase; GGT, γ-glutaminase.

Supplementary: Table 5 Filtering subsets of variables based on the importance of features of random forest

Variable	Variable type	Variable	Variable type
Age	Continuous	TC	Continuous
SBP	Continuous	HDL-C	Continuous
Hypertension	Classification	TB	Continuous
BPcate	Classification	aUA	Continuous
ALB	Continuous	LDL-C	Continuous
SD	Continuous	AIP	Continuous
DBP	Continuous	TP	Continuous
FBG	Continuous	LAP	Continuous
LpH	Continuous	GGT	Continuous
BMI	Continuous	AST	Continuous

CR	Continuous	HBDH	Continuous
Height	Continuous	LCI	Continuous
TyG	Continuous	LDH	Continuous
WHR	Continuous	ALP	Continuous
ALT	Continuous	DB	Continuous
GLO	Continuous	AI	Continuous
THT	Continuous	APOB	Continuous
BAI	Continuous		

Abbreviations: SBP, systolic blood pressure; BPcate, blood pressure classification; ALB, albumin; SD, pulse pressure difference; DBP, diastolic blood pressure; FBG, fasting blood glucose; LpH, low-high-density lipoprotein ratio; BMI, body mass index; CR, creatinine; TyG, triglyceride blood glucose index; WHR, waist-to-height ratio; ALT, alanine aminotransferase; GLO, globulin; THT, bilirubin comprehensive index; BAI, body obesity index; TC, total cholesterol; HDL-C, high-density lipoprotein cholesterol; TB, total bilirubin; aUA, uric acid; LDL-C, low-density lipoprotein cholesterol; AIP, Plasma arteriosclerosis index; TP, total protein; LAP, fat accumulation product index; GGT, γ-glutaminase; AST, aspartate aminotransferase; HBDH, α-hydroxybutyrate dehydrogenase; LCI, blood lipid index; LDH, lactate dehydrogenase; ALP, alkaline phosphatase; DB, direct bilirubin; AI, arteriosclerosis index; APOB apolipoprotein B.

Supplementary: Table 6 Selection of variable subsets based on random forest's ranking importance

Variable	Variable type	Variable	Variable type
HBDH	Continuous	GLO	Continuous
SD	Continuous	ALP	Continuous
LDH	Continuous	THT	Continuous
aUA	Continuous	AI	Continuous
BMI	Continuous	FHDM	Classification
LCI	Continuous	fhstroke	Classification
WHR	Continuous	fhchd	Classification
BAI	Continuous	Minority	Classification
TyG	Continuous	Sex	Classification
LAP	Continuous	Career	Classification
GGT	Continuous	Height	Continuous
TB	Continuous	AIP	Continuous
DB	Continuous	ALB	Continuous
AST	Continuous	CR	Continuous
APOB	Continuous	APOAB	Continuous

Abbreviations: HBDH, α-hydroxybutyrate dehydrogenase; SD, pulse pressure difference; LDH, lactate dehydrogenase; aUA, uric acid; BMI, body mass index;; LCI, blood lipid index;; WHR, waist-to-height ratio; BAI, body obesity index;TyG, triglyceride blood glucose index; LAP, fat accumulation product index; GGT, γ-glutaminase; TB, total bilirubin; DB, Direct Bilirubin; AST, aspartate aminotransferase;APOB apolipoprotein B; GLO, globulin; ALP, alkaline phosphatase; THT, bilirubin comprehensive index; AI,

arteriosclerosis index; FHDM,Family history of diabetes; Fhstroke,Family history of stroke; Fhchd,Family history of coronary heart disease; AIP, Plasma arteriosclerosis index; CR, creatinine; APOAB, apolipoprotein AB.

Based on Logistic regression algorithm forward biased likelihood estimation method (FLR) and Lasso regression and random forest (RF) characteristic importance (CI) and permutation importance (PI) methods to screen variables .Use the filtered variables to create a subset of variables.Combine the selected variable subset and the full variable subset with the Adaboost algorithm to establish a prediction model.The parameter values of the model are shown in Table 7.1

Supplementary: Table 7.1 Hyperparameter values of the Adaboost model under different variable subsets

Model	Parameter categories	
	learning_rate	n_estimators
Lasso	0.016	223
CI	0.062	869
PI	0.066	165
FLR	0.066	868
All	0.131	868

Based on Logistic regression algorithm forward biased likelihood estimation method (FLR) and Lasso regression and random forest (RF) characteristic importance (CI) and permutation importance (PI) methods to screen variables .Use the filtered variables to create a subset of variables.Combine the selected variable subset and the full variable subset with the support vector machine (SVM) algorithm to establish a prediction model.The parameter values of the model are shown in Table 7.2.

Supplementary: Table 7.2 Hyperparameter values of the SVM model under different variable subsets

Model	Parameter categories		
	C	gamma	kernel
Lasso	10	0.001	rbf
CI	1	0.001	rbf
PI	10	0.001	rbf
FLR	10	-	linear
ALL	100	0.0001	rbf

Based on Logistic regression algorithm forward biased likelihood estimation method (FLR) and Lasso regression and random forest (RF) characteristic importance (CI) and permutation importance (PI) methods to screen variables .Use the filtered variables to create a subset of variables.Combine the selected variable subset and the full variable subset with the support vector machine (SVM) algorithm to establish a prediction model.The parameter values of the model are shown in Table 7.3.

Supplementary: Table7.3 Hyperparameter values of the RF model under different variable subsets

Model	Parameter categories				
	max_depth	max_features	min_samples_leaf	min_samples_split	n_estimators
Lasso	8	0.45	11	12	299
CI	12	0.33	19	16	235
PI	14	0.29	11	12	462
FLR	9	0.37	11	15	298
ALL	10	0.37	15	16	298

Based on Logistic regression algorithm forward biased likelihood estimation method (FLR) and Lasso regression and random forest (RF) characteristic importance (CI) and permutation importance (PI) methods to screen variables .Use the filtered variables to create a subset of variables.Combine the selected variable subset and the full variable subset with the L1 regularised logistic regression (L1-LR) algorithm to establish a prediction model.The parameter values of the model are shown in Table 7.5.

Supplementary: Table7.4 Hyperparameter values of the F1-LR model under different variable subsets

Model	Parameter categories
	C
lasso	0.100
CI	0.001
PI	1.000
FLR	0.100
ALL	0.100

Based on Logistic regression algorithm forward biased likelihood estimation method (FLR) and Lasso regression and random forest (RF) characteristic importance (CI) and permutation importance (PI) methods to screen variables .Use the filtered variables to create a subset of variables.Combine the selected variable subset and the full variable subset with L1 regularised logistic regression (L1-LR), random forest (RF), support vector machine (SVM), and Adaboost algorithm to establish a prediction model, Area Under Curve(ACU) values of different models on training and test sets see Table 8 for details.

Supplementary: Table 8 AUC values of different models on training and test sets

Model	AUC		Model	AUC	
	Training sets	Test sets		Training sets	Test sets
Lasso-L1-LR	0.805	0.810	lasso-SVM	0.804	0.810
CI-L1-LR	0.796	0.801	CI-SVM	0.794	0.802
PI-L1-LR	0.731	0.742	PI-SVM	0.731	0.775
FLR-L1-LR	0.807	0.817	FLR-SVM	0.806	0.814
ALL-L1-LR	0.806	0.811	ALL-SVM	0.800	0.804
Lasso-Adaboost	0.793	0.798	lasso-RF	0.800	0.803
CI-Adaboost	0.749	0.796	CI-RF	0.800	0.805
PI-Adaboost	0.728	0.750	PI-RF	0.740	0.763
FLR-Adaboost	0.790	0.798	FLR-RF	0.802	0.804
ALL-Adaboost	0.785	0.805	ALL-RF	0.802	0.803

Based on Logistic regression algorithm forward biased likelihood estimation method (FLR) and Lasso regression and random forest (RF) characteristic importance (CI) and permutation importance (PI) methods to screen variables .Use the filtered variables to create a subset of variables.Combine the selected variable subset and the full variable subset with the Adaboost algorithm to establish a prediction model, and select the optimal prediction model of the algorithm by comprehensively comparing the degree of discrimination and calibration between the models. See Table 9.1 for details.The optimal prediction model of the Adaboost algorithm is established based on the subset of variables selected by Lasso.

Supplementary: Table 9.1 Comparison of prediction performance of different variable subsets based on Adaboost algorithm

	AUC	Youden Index	Optimal threshold	Sensitivity(%)	Specificity (%)	PPV(%)	NPV(%)	Proportion of high-risk population(%)	Brier Score	Homser-Lemeshow χ^2	*P* Value
CI	0.796(0.780,0.811)	0.482	0.09	77.11	71.14	22.5	96.6	34.5	0.078(0.071,0.086)	26.61	<0.01
FLR	0.798(0.782,0.814)	0.503	0.09	82.73	67.55	21.7	97.3	37.4	0.078(0.070,0.085)	29.83	<0.01
PI	0.750(0.733,0.767)	0.382	0.09	67.47	70.74	20.0	95.2	31.6	0.082(0.074,0.091)	23.20	<0.01
Lasso	0.798(0.782,0.813)	0.472	0.11	73.09	74.10	23.5	96.2	30.4	0.078(0.070,0.086)	13.81	0.09
all	0.805(0.789,0.820)	0.519	0.11	74.30	77.55	26.5	96.5	29.1	0.078(0.070,0.086)	23.52	<0.01

Abbreviations:AUC,area under the receiver operating characteristic curve; PPV,positive predictive value; NPV,negative predictive value**;**FLR,forward biased likelihood estimation;CI,characteristic importance;PI,permutation importance

10.3389/fcvm.2022.854287

Based on Logistic regression algorithm forward biased likelihood estimation method (FLR) and Lasso regression and random forest (RF) characteristic importance (CI) and permutation importance (PI) methods to screen variables .Use the filtered variables to create a subset of variables.Combine the selected variable subset and the full variable subset with the Logistic regression algorithm to establish a prediction model, and select the optimal prediction model of the algorithm by comprehensively comparing the degree of discrimination and calibration between the models. See Table 9.2 for details.The optimal prediction model of the LR algorithm is established based on the subset of variables selected by Logistic regression forward biased likelihood estimation method.

Supplementary: Table 9.2 Comparison of prediction performance of different variable subsets based on L1-LR algorithm

	AUC	Youden Index	Optimal threshold	Sensitivity(%)	Specificity (%)	PPV(%)	NPV(%)	Proportion of high-risk population(%)	Brier Score	Homser-Leme show $\chi 2$	*P* Value
CI	0.801(0.785,0.816)	0.477	0.09	73.09	74.63	23.9	96.2	31.5	0.078(0.070,0.086)	10.14	0.26
FLR	0.817(0.801,0.832)	0.524	0.11	73.49	78.86	27.4	96.5	26.7	0.076(0.069,0.084)	11.51	0.17
PI	0.742(0.725,0.759)	0.361	0.08	74.70	61.35	17.4	95.7	41.2	0.081(0.073,0.090)	10.09	0.26
Lasso	0.810(0.794,0.825)	0.489	0.08	79.92	69.00	21.9	96.9	36.0	0.077(0.069,0.085)	10.64	0.22
all	0.811(0.801,0.832)	0.493	0.09	77.91	71.44	22.9	96.7	32.5	0.077(0.069,0.085)	11.35	0.18

Abbreviations:L1-LR,L1 regularised logistic regression;AUC,area under the receiver operating characteristic curve; PPV,positive predictive value; NPV,negative predictive value;FLR,forward biased likelihood estimation;CI,characteristic importance;PI,permutation importance

Based on Logistic regression algorithm forward biased likelihood estimation method (FLR) and Lasso regression and random forest (RF) feature importance (CI) and permutation importance (PI) methods to screen variables .Use the filtered variables to create a subset of variables.Combine the selected variable subset and the full variable subset with the RF algorithm to establish a prediction model, and select the optimal prediction model of the algorithm by comprehensively comparing the degree of discrimination and calibration between the models. See Table 9.3 for details.The optimal prediction model of the RF algorithm is established based on the subset of variables selected by Logistic regression forward biased likelihood estimation method.

Supplementary: Table 9.3 Comparison of prediction performance of different variable subsets based on RF algorithm

	AUC	Youden Index	Optimal threshold	Sensitivity(%)	Specificity (%)	PPV(%)	NPV(%)	Proportion of high-risk population(%)	Brier Score	Homser-Lemeshow χ^2	*P* Value
CI	0.805(0.789,0.820)	0.496	0.09	73.90	75.68	24.8	96.4	28.9	0.077(0.070,0.086)	22.81	<0.01
FLR	0.804(0.788,0.820)	0.506	0.08	79.52	71.09	23.0	97.0	33.1	0.077(0.070,0.086)	11.59	0.17
PI	0.763(0.746,0.779)	0.413	0.08	75.90	65.41	19.2	96.0	35.9	0.081(0.073,0.090)	32.75	<0.01
Lasso	0.803(0.787,0.818)	0.482	0.08	75.90	72.31	23.0	96.5	32.5	0.076(0.069,0.085)	11.33	0.18
all	0.803(0.787,0.818)	0.498	0.08	75.10	74.72	24.4	96.5	30.8	0.079(0.071,0.088)	34.44	<0.01

N**Abbreviations:**AUC,area under the receiver operating characteristic curve; PPV,positive predictive value; NPV,negative predictive value;FLR,forward biased likelihood estimation;CI,characteristic importance;PI,permutation importance

Based on Logistic regression algorithm forward biased likelihood estimation method (FLR) and Lasso regression and random forest (RF) feature importance (CI) and permutation importance (PI) methods to screen variables .Use the filtered variables to create a subset of variables.Combine the selected variable subset and the full variable subset with the Support vector machine(SVM) algorithm to establish a prediction model, and select the optimal prediction model of the algorithm by comprehensively comparing the degree of discrimination and calibration between the models. See Table 9.4 for details.The optimal prediction model of the SVM algorithm is established based on the subset of variables selected by Logistic regression forward biased likelihood estimation method.

Supplementary: Table 9.4 Comparison of prediction performance of different variable subsets based on SVM algorithm

	AUC	Youden Index	Optimal threshold	Sensitivity(%)	Specificity (%)	PPV(%)	NPV(%)	Proportion of high-risk population(%)	Brier Score	Homser-Lemeshow $\chi 2$	P Value
CI	0.802(0.786,0.817)	0.483	0.09	76.31	72.01	22.9	96.5	32.3	0.077(0.069,0.086)	14.97	0.06
FLR	0.814(0.798,0.829)	0.511	0.11	73.90	77.16	26.0	96.5	38.4	0.076(0.069,0.084)	16.10	0.04
PI	0.755(0.738,0.771)	0.371	0.09	69.08	67.99	19.0	95.3	34.8	0.081(0.072,0.089)	13.04	0.11
Lasso	0.810(0.794,0.825)	0.487	0.09	77.91	70.79	22.5	96.7	32.8	0.077(0.069,0.085)	11.53	0.17
all	0.804(0.788,0.820)	0.481	0.09	75.10	73.01	23.2	96.4	32.3	0.077(0.070,0.085)	8.16	0.42

Abbreviations:AUC,area under the receiver operating characteristic curve; PPV,positive predictive value; NPV,negative predictive value;FLR,forward biased likelihood estimation;CI,characteristic importance;PI,permutation importance
